# Morphological, lytic, and genetic characteristics of three *Brucella* phages isolated from Inner Mongolia Autonomous Region

**DOI:** 10.3389/fmicb.2025.1550801

**Published:** 2025-04-30

**Authors:** Yu Zhang, Dongri Piao, Qingqing Xu, Yu Fan, Hongyan Zhao, Kun Li, Guozhong Tian, Kuo Han, Hai Jiang

**Affiliations:** ^1^National Key Laboratory of Intelligent Tracking and Forecasting for Infectious Diseases, National Institute for Communicable Disease Control and Prevention, Chinese Center for Disease Control and Prevention, Beijing, China; ^2^Chaoyang Center for Disease Control and Prevention, Beijing, China

**Keywords:** *Brucella*, *Brucella* phage, phylogenetic tree, whole-genome analysis, diversity

## Abstract

This study comprehensively examined three *Brucella* phages (A1, NMY-1, and NMY-2) isolated from Inner Mongolia Autonomous Region. Electron microscopy classified them as short-tailed phages. A1 and NMY-1 lysed smooth strains of *Brucella abortus*, *Brucella melitensis*, and *Brucella suis*, while NMY-2 lysed rough strains of *Brucella melitensis* and *Brucella canis*. The optimal multiplicity of infection for A1, NMY-1, and NMY-2 was lower than that of Tb_C_. A1 and NMY-2 had short growth cycles, and NMY-1 had a long one. All three phages showed high stability against temperature, pH, and ultraviolet exposure. Their genomes were double-stranded DNA, about 38 kb long with a 48% GC content. For each phage, 53 genes were predicted, with no drug-resistance, virulence, or lysogenic genes identified. SNP and InDel analysis revealed significant differences in genes encoding hypothesized tail-collar proteins. Based on SNP data, the phylogenetic tree indicated that phage Bk_W_ (GenBank: KC556893) was the closest relative of A1, NMY-1, and NMY-2. These findings significantly enhance our understanding of *Brucella* phage diversity, which is crucial for developing phage-based biocontrol strategies. The host-lysis spectra can guide the selection of effective phages for treating *Brucella* infections. The absence of harmful genes makes these phages potential safe candidates for phage therapy. Moreover, the genetic and phylogenetic insights support further research on phage evolution and classification.

## 1 Introduction

*Brucella* species are Gram-negative bacteria and the causative agent of brucellosis, a bacterial zoonosis that exists worldwide (Issabekov et al., 2022; Zhang N. et al., 2018). The World Health Organization estimated that new cases of brucellosis exceed 2.1 million per year globally (Laine et al., 2023), and brucellosis has become a serious public health problem worldwide. *Brucella* infection may lead to reproductive disorders and abortion in animals. In humans, the acute phase of infection is characterized by fever, weakness, excessive sweating, muscle pain, and enlargement of the liver, spleen, and lymph nodes, while the chronic phase is characterized by joint pain (Zhang H.-X. et al., 2018). The non-specific presentation of brucellosis makes it difficult to diagnose and treat the disease in a timely manner. Identification of *Brucella* species requires a combination of identification methods such as serum agglutination, dye sensitivity, and phage lysis (Corbel, 2006). In addition, several molecular assays are available for further speciation, such as PCR for BCSP31, 16S rRNA genes, and IS711 elements (Baily et al., 1992; Hinić et al., 2008) and real-time fluorescent PCR (Lopez-Goñi et al., 2008; Mayer-Scholl et al., 2010). However, existing molecular typing systems do not cover all known species and biological variants of the genus *Brucella*, but phages have specific lysis of their host bacteria, so the use of *Brucella* phages is an indispensable part of the process of identification and detection of *Brucella*.

A total of 12 *Brucella* species have been isolated and identified and the predominant species in China is *Brucella melitensis* (Cui and Jiang, 2018; Hull and Schumaker, 2018). *Brucella* phages are viruses that can infect *Brucella* and exhibit host specificity (Farlow et al., 2014). Since the discovery of the *Brucella* phage Tb (Tbilisi) in the 1960s, *Brucella* phages have been used for identification or typing of *Brucella* (Hammerl et al., 2014). Based on the host range, *Brucella* phages are classified into six groups (Zhu et al., 2009): Tbilisi (Tb), Firenze (Fz), Weybridge (Wb), Berkeley (Bk), R/C, and Izatnagar (Iz). These *Brucella* phages have a very similar morphology and genomic structure, but they differ in their host specificity (Cui et al., 1992; Hammerl et al., 2017; Mohan and Saxena, 2020; Projahn et al., 2020; Sergueev et al., 2017). Previously, the *Brucella* phages A1, NMY-1, and NMY-2 were isolated from Inner Mongolia Autonomous Region by Cui et al. (1995). In this study, the three phages were analyzed in detail for their morphological features, lysis profile, optimal multiplicity of infection (MOI), one-step growth curve, thermal stability, UV stability, and acid–base stability. In addition, for the first time, whole-genome sequencing of these *Brucella* phages was performed. This study provides in-depth data that are important for understanding the population structure of *Brucella* phages, identifying and classifying *Brucella* phages, and improving the diagnosis and treatment of brucellosis.

## 2 Materials and methods

### 2.1 Bacterial strains and phages

The *Brucella* strains used in this study were selected from the *Brucella* library constructed by the *Brucella* Disease Unit of the Institute of Infectious Disease Prevention and Control of the Chinese Center for Disease Control and Prevention. The 71 selected strains included 11 *Brucella abortus* strains, 10 *Brucella suis* strains, 13 *Brucella canis* strains, and 37 *Brucella melitensis* strains (Supplementary Table S1). *Brucella* phages A1, NMY-1, and NMY-2 were obtained from the Institute of Endemic Disease Control of Inner Mongolia Autonomous Region; Tb_C_, Wb_C_, and Iz_C_ were obtained from WOAH Reference Laboratory for Brucellosis, UK; and Bk2_C_ and R_C_ were obtained from the Institute of Epidemiology, Beijing (Table 1). All these brucellaphages were propagated and analyzed at Infectious Disease Prevention and Control of the Chinese Center for Disease Control and Prevention (herein the CCDC propagated phage is designated Tb_C_, Wb_C_, Iz_C_, Bk2_C_, and R_C_).

**Table 1:** *Brucella* phage sources and proliferating strains.

Group	Brucella phage	Source	Proliferating strain
1	Tb_C_	WOAH Reference Laboratory for Brucellosis, United Kingdom	544A
3	Wb_C_	WOAH Reference Laboratory for Brucellosis, United Kingdom	S1330
4	Bk2_C_	The Institute of Epidemiology, Beijing	Isfahan
5	R_C_	The Institute of Epidemiology, Beijing	45/20
6	Iz_C_	WOAH Reference Laboratory for Brucellosis, United Kingdom	B115
	A1	Institute of Endemic Disease Control of Inner Mongolia Autonomous Region	S19
	NMY-1	Institute of Endemic Disease Control of Inner Mongolia Autonomous Region	Isfahan
	NMY-2	Institute of Endemic Disease Control of Inner Mongolia Autonomous Region	45/20

### 2.2 Bacteriophage purification and proliferation

The phages were purified by using soft agar overlays (Rigby et al., 1989). Briefly, single phage plaque with neat and translucent edges were picked up, immersed in Brønsted broth medium, and centrifuged at 8,000 rpm for 5 min. The supernatant was filtered through a 0.22-μm microporous membrane, and the above steps were repeated three to five times until the plaque were of uniform size and morphology. The phage suspension was subjected to gradient dilution and filtered through a 0.22-μm microporous membrane, and it was stored in a 4°C refrigerator after determination of phage titer via soft agar overlays (Rigby et al., 1989).

### 2.3 Morphological analysis

The phosphotungstic acid negative staining method, as described by Hagens and Loessner (2007) and Hastings and Gibson (1963), was employed. A phage suspension with a concentration of 10^8^ plaque-forming units (PFU)/mL was carefully dropped onto copper grids. After 5 min, excess suspension was blotted off with filter paper. Next, 2% phosphotungstic acid staining solution was added dropwise for 1 min, and excess staining solution was blotted off with filter paper again. Phage morphology was observed under a Tecnai12 transmission electron microscope (FEI, Eindhoven, Netherlands) after drying at room temperature.

### 2.4 Host range analysis

A total of 71 *Brucella* strains were tested using the bilayer agar method and the spotting method to determine the lytic range of the phages (Hammerl et al., 2017). Each *Brucella* strain [10^9^ colony-forming units (CFU)/mL] was added to the melted semi-solid medium, which was mixed and poured into a flat dish containing a bottom layer of *Brucella* agar medium. After solidification, each dilution of phage was dropped at different positions, left to dry, and incubated in a constant temperature incubator at 37°C for 48 h. The surface of the medium was then observed for the presence or absence of phage plaques.

### 2.5 Calculation of optimal multiplicity of infection

The concentration of host bacteria was adjusted to a turbidity of 1.0 mcf and diluted serially to achieve a bacterial titer of 1 × 10^7^ CFU/mL. The phage and host bacteria were mixed in equal volumes at MOIs of 10, 1, 0.1, 0.01, and 0.001, and incubated in an air shaker at 37°C for 24 h at 200 r/min. The phage suspension was obtained by filtration through a 0.22-μm microporous filter membrane. The titer was determined by the double-agar plate method, and the ratio corresponding to the highest titer was considered as the MOI of the phage for infection of the host bacteria (Yuan et al., 2020).

### 2.6 One-step growth curve analysis

The one-step growth curve of the phage was determined with reference to previous methods with some modifications (Ellis and Delbruck, 1939). The host bacteria and phage were mixed in equal volumes based on the MOIs of the phages. This was followed by adsorption at 37°C for 10 min and centrifugation at 4°C for 15 min at 5,500 rpm. The supernatant was discarded, and the precipitate was resuspended in 10 mL of liquid medium that was incubated in an air shaker at 37°C and 200 rpm for 2–24 h. Phage titer was measured by sampling at different time points between 2 and 24 h. A one-step growth curve was drawn with time as the horizontal coordinate and measured titer as the vertical coordinate.

### 2.7 Physicochemical stability analysis

The temperature, UV, and pH sensitivity of the phages was determined by exposing them to different settings. EP tubes with phage suspension were placed in a constant temperature water bath at 40°C, 50°C, 60°C, 70°C, or 80°C for 2 h to determine phage titer at these temperatures. A curve was plotted with temperature as the horizontal coordinate and phage titer as the vertical coordinate. To determine the effect of UV irradiation at different times, the phage suspension was spread on a prepared Petri dish and placed at the center of a biosafety cabinet [50 cm vertical distance from the ultraviolet light source, ultraviolet lamp (power 30 W) in the cabinet]. Samples were obtained at 3-min intervals from 0 to 30 min, and their titer was measured. For assessing sensitivity to different pH values, the phage suspension was mixed with equal volume of liquid medium in phosphate-buffered saline (PBS). The pH of PBS was adjusted by using HCl and NaOH, and the pH of PBS was determined by a pH meter. The pH value was 2, 4, 6, 8, 10, 12, and 14, respectively. The mixed suspension was placed in a constant temperature incubator (37°C) for 2 h, and phage titer was detected after incubation. A curve was plotted with pH as the horizontal coordinate and phage titer as the vertical coordinate.

### 2.8 Phage DNA extraction and sequencing

Phage genomic DNA was extracted using the *λ* Phage DNA Extraction Kit (Beijing Abiogen Biotechnology Co., Ltd.), according to the manufacturer’s instructions. The phage genome was sequenced using the Illumina Library Prep Kit (DNA Library Prep Kit, San Diego, CA, United States) and the Illumina NovaSeq PE150 Sequencing Platform with >400-fold sequencing coverage. The raw data obtained after sequencing were subject to quality control using the Soapnuke (v2.0.5) software, and *de novo* assembly was performed using the Megahit software (Chen et al., 2018).

### 2.9 Genome analysis

The corresponding annotation information was obtained according to the predicted genes of Tb_M_. The NR, UniProtKB and Swiss-Prot universal functional databases were used to annotate the gene functions (Hulo et al., 2011). Gene prediction of the phage genome was performed using the MetaGeneMark (v3.38) software, with filtration of sequences with a gene nucleic acid length less than 150 bp (Zhu et al., 2010). Virulence genes and drug resistance genes were predicted by VFDB and CARD respectively (Dong et al., 2022). The protein sequences of the predicted genes were used to obtain functional information about the virus from the protein domain database using BLASTP.^1^ The comparative genetic map was constructed using Easyfig 2.2.5 (Sullivan et al., 2011). Using the BLAST (v2.9.0+) software (Camacho et al., 2009), the obtained contigs were compared with the virus database (from the NT database). The NUCmer version 3.1 software was used for comparison, and the MUMmer-4.0.0 software (Marçais et al., 2018) was used to detect SNPs and InDels, with the reference phage Tb_M_ (JN939331). The Neighbor-Joining (NJ) phylogenetic tree was constructed using SNPs, and the phylogenetic tree was visualized with the MEGA software to generate a phylogenetic tree with 1,000 bootstrap replicates (Tamura et al., 2021).

### 2.10 Statistical analysis

Experiments concerning MOI, one-step growth curves and stability were repeated three times per group. The data were statistically analyzed with Excel 2019, presented as mean ± SD, and then plotted using Origin2022 software.

## 3 Results

### 3.1 Phage plaque features and phage ultrastructure

The host bacterium of phages A1, NMY-1, NMY-2, and Tb_C_ are *Brucella abortus* S19, *Brucella melitensis* Isfahan, *Brucella canis* 45/20, and *Brucella abortus* S19, respectively.

**Figure 1:**
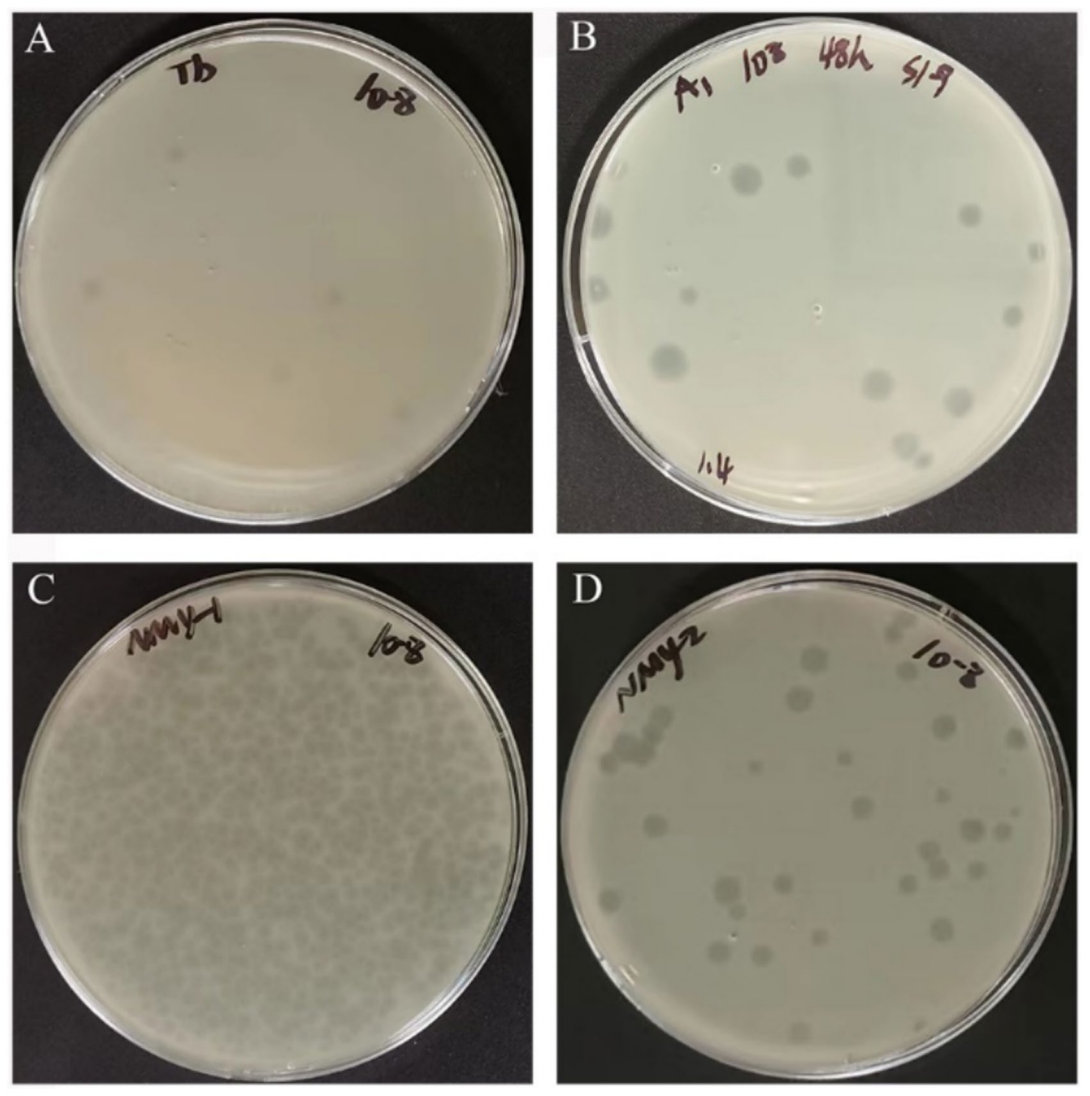
Phage plaque morphology of *Brucella* phages Tb_C_ (host for *B. abortus* S19) **(A)**, A1 (host for *B. abortus* S19) **(B)**, NMY-1 (host for *B. melitensis* Isfahan) **(C)**, and NMY-2 (host for *B. canis* 45/20) **(D)**.

The purified phages formed clear, translucent phage plaques with neat edges on soft agar overlays (Figure 1). The diameters of the A1, NMY-1, NMY-2, and Tb_C_ phage plaques were 5.1 ± 0.8 mm, 2.5 ± 0.4 mm, 3.5 ± 0.4 mm, and 4.1 ± 0.8 mm, respectively. The titers of A1, NMY-1, NMY-2, and Tb_C_ were higher than 10^9^ PFU/mL, as measured by using soft agar overlays.

**Figure 2:**
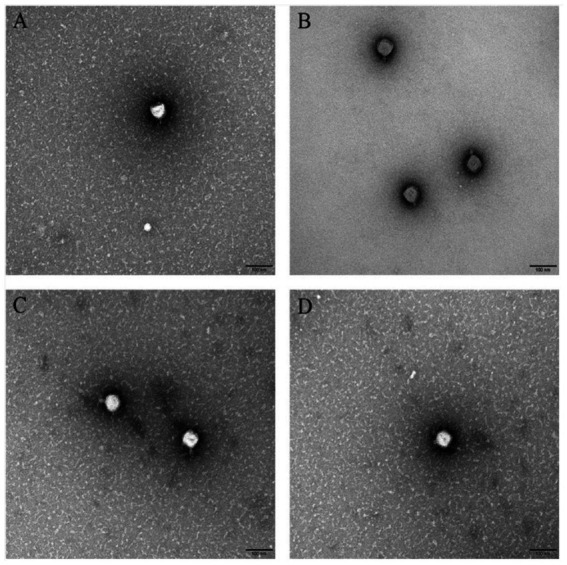
Transmission electron micrograph of *Brucella* phages Tb_C_ (with the host *B. abortus* S19) **(A)**, A1 (with the host *B. abortus* S19) **(B)**, NMY-1 (with the host *B. melitensis* Isfahan) **(C)**, and NMY-2 (with the host *B. canis* 45/20) **(D)**. The scale bar represents 100 nm.

Transmission electron microscopy showed that the phage head was icosahedral and had a short tail (Figure 2). The head diameters of A1, NMY-1, NMY-2, and Tb_C_ were 59.7 ± 1.1 nm, 63.5 ± 0.6 nm, 56.7 ± 0.5 nm, and 57.3 ± 0.2 nm, respectively, and their tail lengths were 15.5 ± 0.3 nm, 14.7 ± 0.5 nm, 14.4 ± 0.6 nm, and 14.8 ± 0.1 nm, respectively.

### 3.2 Host range

Bacteriophages are used at two different concentrations, namely, routine test dilution (RTD) and 10^4^ × RTD, for the identification of *Brucella* (Li et al., 1985). Multiple phage combinations can be used to identify *Brucella* species (Cui et al., 1995; Huangjian, 1980; Mohan and Saxena, 2020; Zhu, 2009). Phage A1 was able to lyse smooth *B. abortus* (10/11), *B. melitensis* (8/24), and *B. suis* (10/10) at RTD. At 10^4^ × RTD, it was able to lyse a larger number of smooth *B. melitensis* (9/24), but showed the same lytic ability for the other two phages. Phage NMY-1 was able to lyse smooth *B. abortus* (10/11), *B. melitensis* (6/24), and *B. suis* (8/10) at RTD. When its concentration was increased to 10^4^ × RTD, it was able to lyse a larger number of smooth *B. melitensis* (16/24) and *B. suis* (10/10), but its lytic effect on smooth *B. abortus* was the same. Phage NMY-2 was able to lyse rough *B. melitensis* (7/13) and *B. canis* (13/13) at RTD, but at 10^4^ × RTD, it was able to lyse all the rough *Brucella* organisms of both bacterial species (Table 2).

**Table 2:** Lytic ability of *Brucella* phages A1, NMY-1, and NMY-2.

Phage	Titer	Smooth *B. abortus* (*n* = 11)	Smooth *B. suis* (*n* = 10)	Smooth *B. melitensis* (*n* = 24)	Rough *B. melitensis* (*n* = 13)	Rough *B. canis* (*n* = 13)
A1	RTD	10	10	8	0	0
	10^4^ × RTD	10	10	9	0	0
NMY-1	RTD	10	10	6	0	0
	10^4^ × RTD	11	10	16	0	0
NMY-2	RTD	0	0	0	7	13
	10^4^ × RTD	0	0	0	13	13
Bk2_C_	RTD	8	7	11	0	0
	10^4^ × RTD	11	10	24	0	0
Wb_C_	RTD	10	10	5	0	0
	10^4^ × RTD	10	10	7	0	0
Tb_C_	RTD	10	1	2	0	0
	10^4^ × RTD	10	10	6	0	0
Iz_C_	RTD	0	0	0	13	13
	10^4^ × RTD	0	1	0	13	13
R_C_	RTD	0	0	0	1	13
	10^4^ × RTD	0	0	0	3	13

### 3.3 Optimal MOI

**Figure 3:**
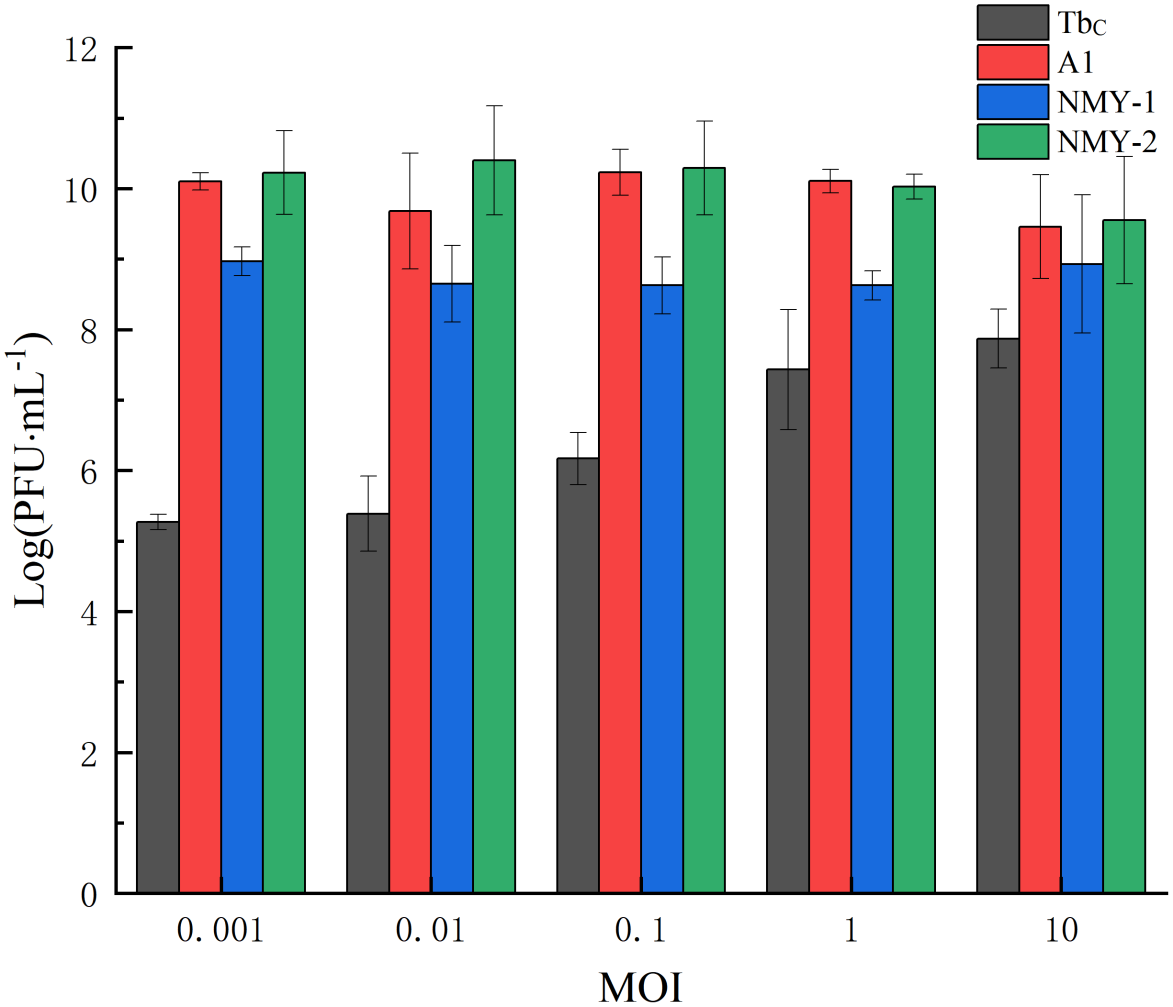
Optimal MOIs of *Brucella* phages Tb_C_ (with the host *B. abortus* S19), A1 (with the host *B. abortus* S19), NMY-1 (with the host *B. melitensis* Isfahan), and NMY-2 (with the host *B. canis* 45/20).

Phage A1 had the highest titer of 1.71 × 10^10^ PFU/mL at an MOI of 0.1 when the host bacterium was *B. abortus* S19. Phage NMY-1 had the highest titer of 9.27 × 10^8^ PFU/mL at an MOI of 0.001 when the host bacterium was *B. melitensis* Isfahan. Phage NMY-2 had the highest titer of 2.51 × 10^10^ PFU/mL at an MOI of 0.01 when the host bacterium was *B. canis* 45/20. The reference phage Tb_C_ had the highest titer of 7.43 × 10^7^ PFU/mL at an MOI of 10 when *B. abortus* 544A was the host bacterium (Figure 3).

### 3.4 One-step phage growth curve

**Figure 4:**
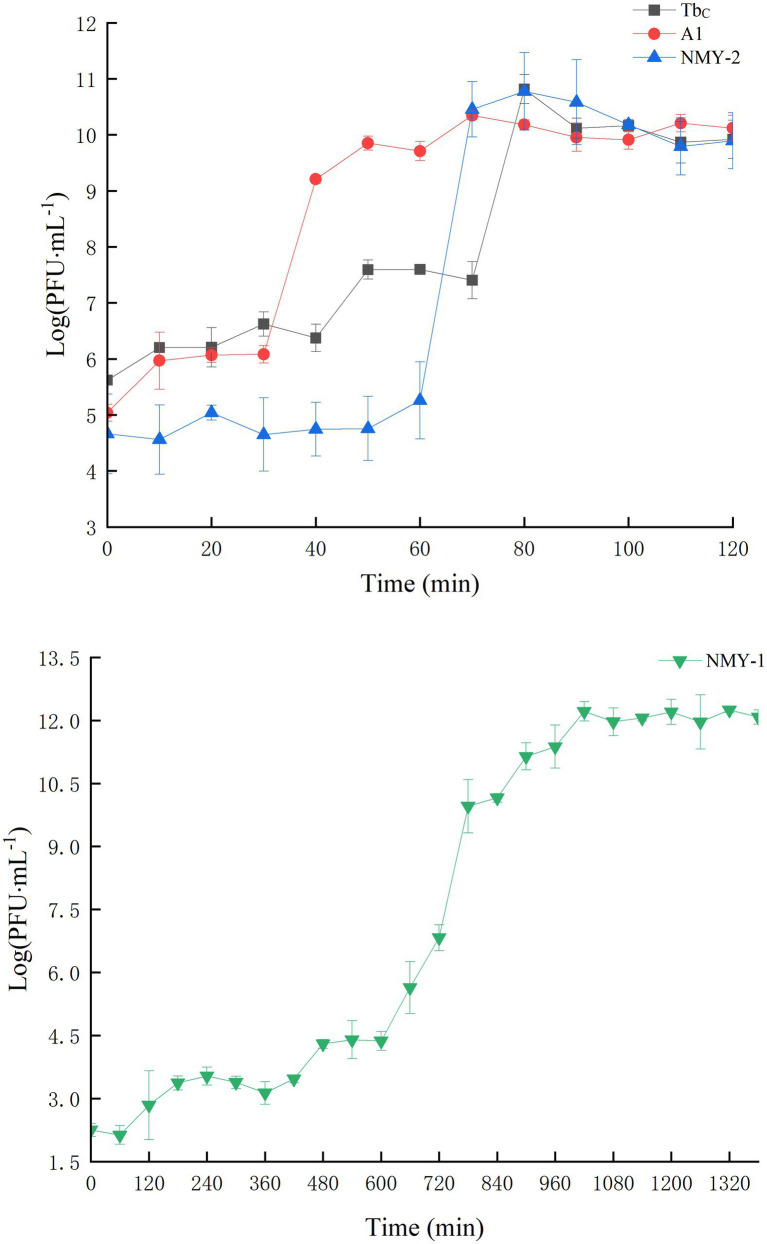
One – step growth curves of *Brucella* phages. TbC, A1, and NMY – 2 (**A**; hosts: *B. abortus* S19 for Tb_C_ and A1, B. canis 45/20 for NMY - 2), NMY – 1 (**B**; host: *B. melitensis* Isfahan).

The phages were mixed with the corresponding host bacteria according to the optimal MOI, incubated with shaking, and sampled once every 10 min to measure phage titer and burst size. The burst size was calculated by dividing the phage titer at the end of lysis by the titer of the host bacteria at the start of infection. The growth curves of Tb_C_, A1, and NMY-2 are shown in Figure 4A, and the growth curve of NMY-1 is shown in Figure 4B. The latent period and eclipse period of Tb_C_ were both 40 min,. and the burst size was 6,800 PFU/cell. The latent period, eclipse period, and burst size of A1 were 30 min, 40 min, and 2,210 PFU/cell, respectively. NMY-2 had a latent period of 60 min, eclipse period of 20 min, and the burst size to be 6,000 PFU/cell. NMY-1 had a latent period of 60 min, eclipse period of 960 min, and the burst size to be 1.90 × 10^5^ PFU/cell.

### 3.5 Physicochemical stability

**Figure 5:**
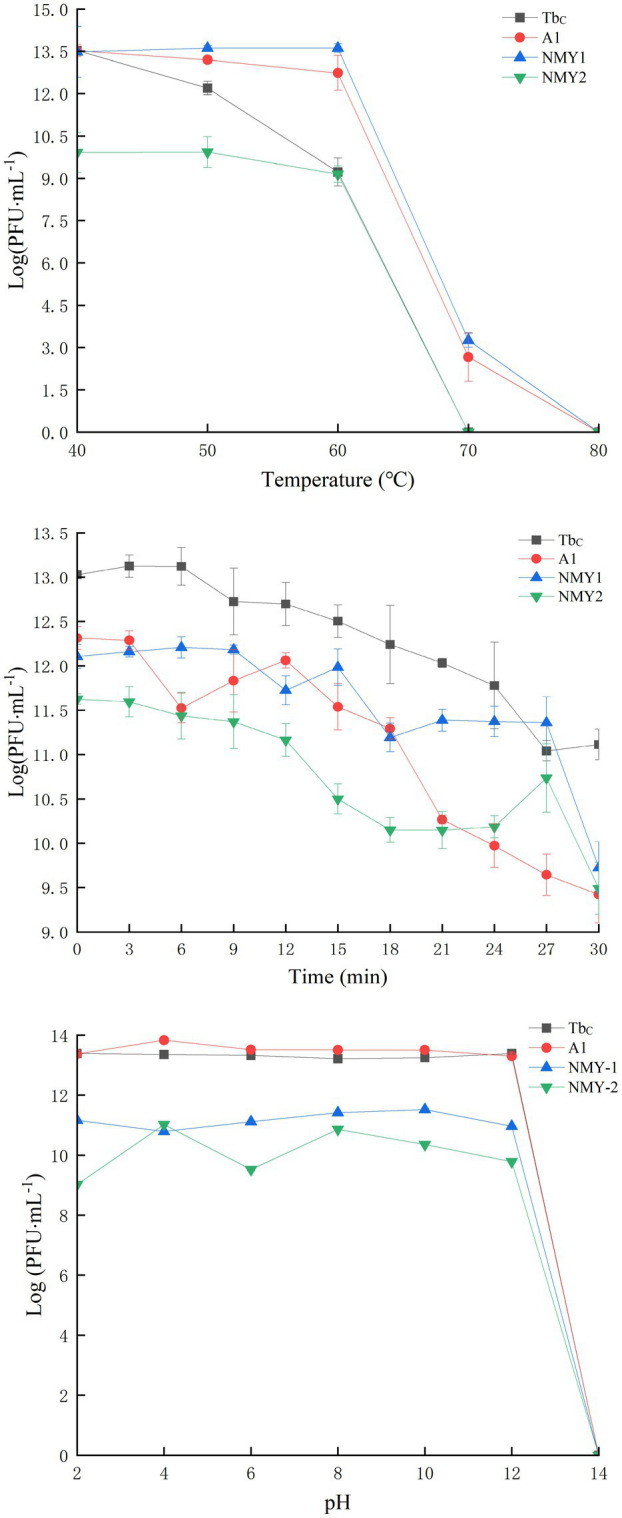
Temperature **(A)**, UV **(B)**, and pH **(C)** stability of the *Brucella* phages Tb_C_ (with the host *B. abortus* S19), A1 (with the host *B. abortus* S19), NMY-1 (with the host *B. melitensis* Isfahan), and NMY-2 (with the host *B. canis* 45/20). The Y-axis shows the logarithm of plaque-forming units per milliliter (PFU/mL).

The phages were alive and stable after treatment at temperatures of 40°C, 50°C, and 60°C for 120 min. When the temperature was increased to 70°C, Phages NMY-2 and Tb_C_ were inactivated, and the titer of NMY-1 and A1 decreased drastically. When the temperature was increased to 80°C, all the phages were inactivated (Figure 5A). The phage titers were also measured after irradiation with UV light. The results showed that the phage titers decreased gradually with time, but they still exhibited high titer after 30 min of irradiation (Figure 5B). With regard to the effect of pH, the phages could not survive in environments of pH 14, but they maintained high activity levels at pH 2–12. These findings show that the phages had good acid and base tolerance (Figure 5C).

### 3.6 Whole-genome analyses

#### 3.6.1 Genome characteristics

The *Brucella* phage A1 genome consists of 38,380 base pairs (bp), with 48.17% GC content. The NMY-1 genome consists of 38,380 bp, with 48.14% GC content, and the NMY-2 genome consists of 38,334 bp, with 48.18% GC content. The average genome size was 38,365 bp, and the average GC content was 48.16%. The number of predicted genes was 53 for all three phages, and the predicted gene length was more than 150 bp. The genomes of the three phages were arranged in a circular pattern and comprised double-stranded DNA. These features are consistent with those of order Arthrophage and the short-tailed phage family.

#### 3.6.2 Genomic comparison and functional annotation

**Figure 6:**
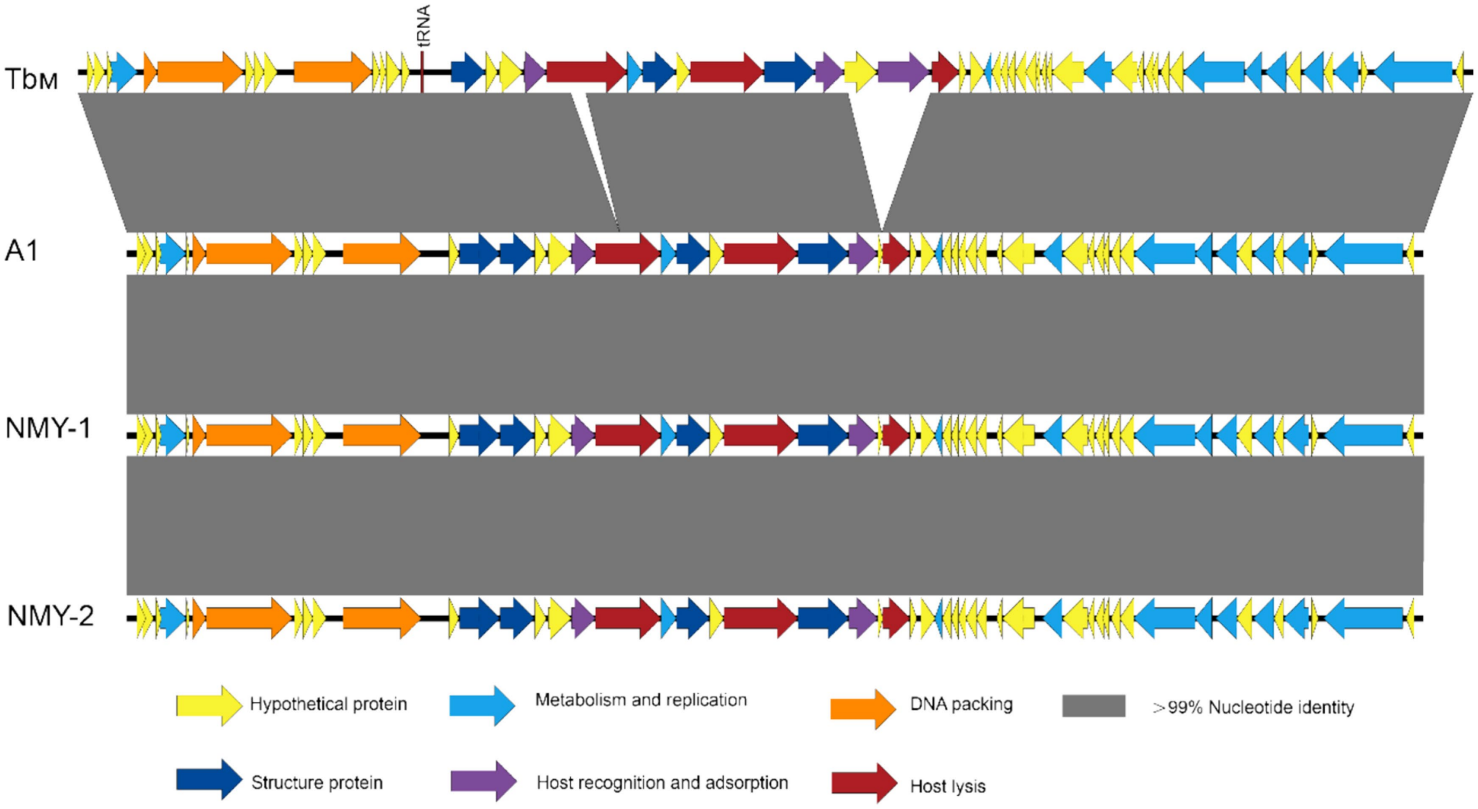
Whole genome alignment of *Brucella* phages A1, NMY-1, and NMY-2, with Tb_M_ (GenBank accession number JN939331) as the reference phage.

The genome sequences of the *Brucella* phages A1, NMY-1, and NMY-2 were compared in the NCBI database separately, and the genomic maps of the three phages are shown in Figure 6 Their genomic structure showed a high degree of similarity. In phage A1, NMY-1, and NMY-2, 22 out of 53 genes were clearly identified as having well-characterized protein-coding functions, while the functions of the remaining 31 genes remained to be determined. The predicted genes were found to be related to DNA replication, DNA metabolism, DNA packaging, host lysis, host recognition and adsorption, and structure. None of the genes were associated with lysogenesis, drug resistance, or virulence. Furthermore, genes encoding putative tail-collar proteins, which are likely to be closely associated with phage host specificity, were identified in phages A1, NMY-1, and NMY-2. Genomic comparison at the DNA level indicated that the genomes of phage A1, NMY-1, NMY-2, and Tb_M_ exhibited more than 93% identity. Moreover, the genome of the Tb_M_ phage presented two large insertions. One was located in the gene encoding amidase, another in the gene encoding a hypothetical protein and putative carbohydrate-binding protein.

### 3.7 SNP/InDel assay of the phages

The three phages showed more than 99.75% nucleotide identity according to genome matching analyses. To compare the nucleotide sequences, the Tb_M_ (JN939331) phage was used as the reference phage, and the comparison results are presented in Supplementary Table S2.

A total of 44 SNPs and 15 InDels were present in the *Brucella* phages. The gene encoding the putative phage tail collar protein showed the most significant variation. The most pronounced insertion in reference phage Tb_M_ relative to A1, NMY-2, and NMY-1 was found in ORF 23—a region encoding a structural protein that has significant homology with the putative protein in other phages and partial homology with the neck protein in tailed phages. In ORF 27, which encodes the putative tail collar protein, 13 amino acid substitutions and one InDel were detected. In our dataset, two SNP sites, positioned at 22,364 and 22,470 within the tail-collar protein gene (ORF 27), exhibited variability across Tb_M_, NMY-1, NMY-2, and A1 *Brucella* phages, consistent with a previous report (Flores et al., 2012). Analogous to the findings in the previous study (Flores et al., 2012), the gene encoding the putative phage tail collar protein demonstrated the highest degree of variability. This is indicative of extensive polymorphism in the gene. In particular, NMY-2 has a major deletion in ORF 27. This might mean that the variation in this region is responsible for the host range of *Brucella* phages. An SNP was detected in ORF 25, which encodes the putative peptidoglycan structural domain associated with host lysis and degradation of peptidoglycans (Flores et al., 2012). In addition, mutations were detected within the gene encoding the putative *Brucella* phage HNH nucleic acid endonuclease (ORF 8), a homing nuclease that plays a role in DNA binding and cleavage and degradation of viral and bacterial nucleic acids and has been detected in all *Brucella* phage genomes to date (Farlow et al., 2014; Flores et al., 2012). In addition, 14 other ORFs (ORFs 2, 12, 13, 14, 16, 17, 19, 20, 21, 30, 43, 46, 52, and 58) showed genetic variations in the three *Brucella* phages.

### 3.8 Phylogenetic tree of the phages

**Figure 7:**
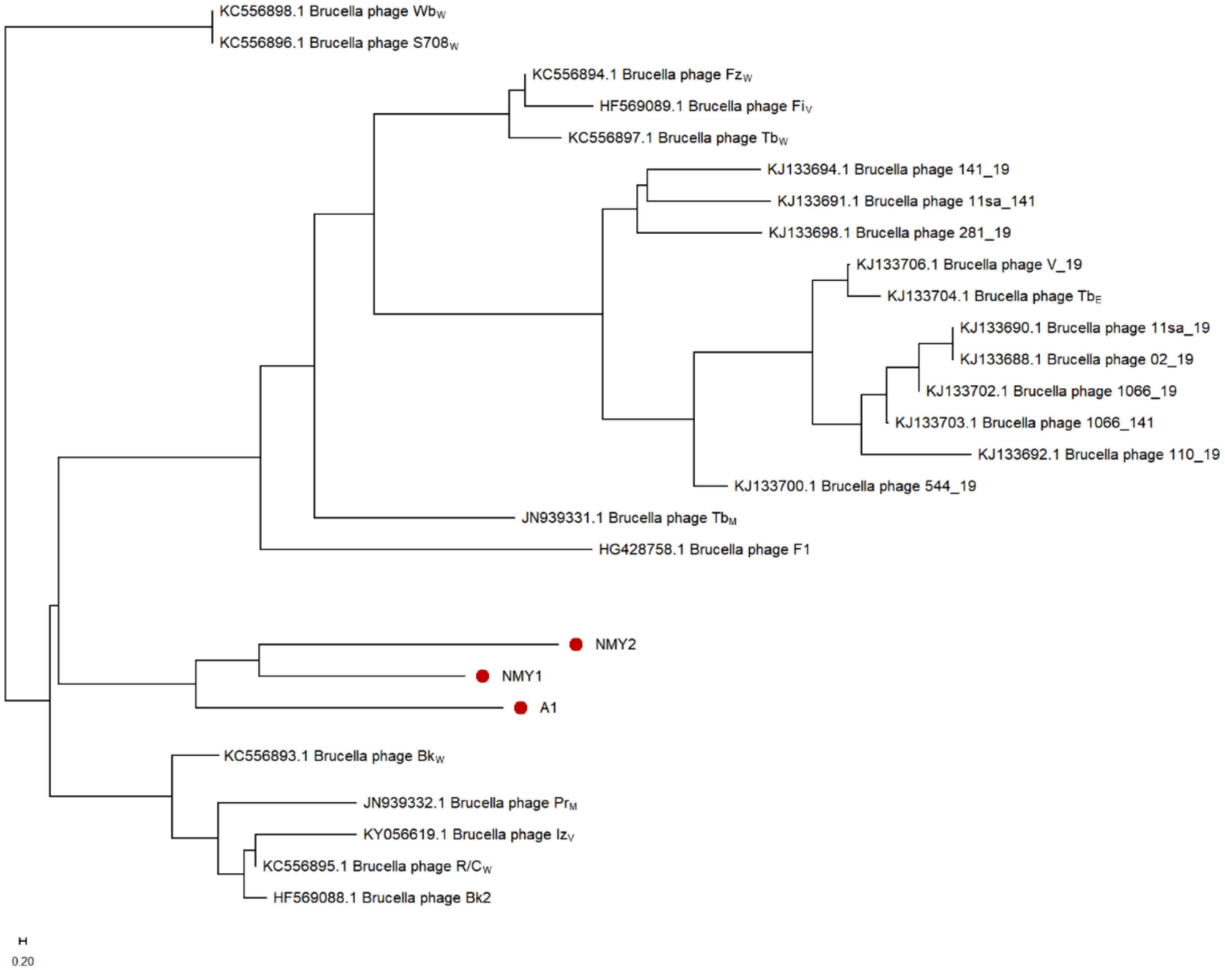
Phylogenetic tree based on the SNPs of the *Brucella* phage genomes.

To further study the genetic relationships of the *Brucella* phages, the NJ phylogenetic tree was constructed based on the detected SNPs and visualized with the MEGA software. The branch lengths in the horizontal direction of the phylogenetic tree represent the genetic distance, with closer distances indicating closer affinity. As shown in Figure 7, *Brucella* phages can be roughly divided into three groups: the first group includes phages Bk_W_ (KC556893), Bk2 (HF569088), R/C_W_ (KC556895), Iz_V_ (KY056619), and Pr (JN939332), which mainly lyse *B. abortus*, *B. melitensis*, and *B. suis*; the second group consists of phages Wb_W_ (KC556898) and S708_W_ (KC556896), which mainly lyse *B. suis* and *B. abortus*; and the remaining phages form the third group, which mainly lyse *B. abortus*. The three *Brucella* phages A1, NMY-1, and NMY-2 isolated from Inner Mongolia Autonomous Region were in the same branch and were closer to the reference phage Bk_W_ (KC556893) than to the other reference phages. The lysis ranges of A1, NMY-1 and Bk_W_ (KC556893) were consistent, which indicates that the grouping results might be related to their host specificity.

## 4 Discussion

This study comprehensively characterizes three *Brucella* phages—A1, NMY-1, and NMY-2—from the Inner Mongolia Autonomous Region and presents detailed data on their morphological characteristics, host range, MOI, growth characteristics, physicochemical characteristics, genomic map, genetic variations, and phylogenetic relationships.

The features of the phage plaques and ultrastructure of the phages were typical of short-tailed *Brucella* phages. The lysis range of A1 and NMY-1 was similar to that of BK2_C_. Based on the observed lysis of smooth *B. abortus*, *B. melitensis*, and *B. suis* at RTD, both phages could be classified in the fourth group of phages according to host specificity. NMY-2 only lysed rough *B. melitensis* and *B. canis*, similar to that of group V phage R, and can therefore be classified in the fifth group of phages. The morphology, host range, and tolerance to chemical and physical factors of NMY-2 are largely consistent with those previously reported for the fifth group of phages (Ackermann et al., 1981; Corbel et al., 1988; Cui et al., 1995; McDuff et al., 1962), but with some differences. However, some of the results for host range differ from those of previous studies. For example, while the present results showed that NMY-1 did not lyse rough *Brucella* and NMY-2 did not lyse smooth *Brucella*, Cui et al. (1995) and Huangjian (1980) reported that NMY-1 and NMY-2 could lyse smooth *B. melitensis*, *B. abortus*, and *B. suis*, as well as rough *B. abortus* and *B. canis*. This difference may be due the “lysis from without” (Abedon, 2011) effect, which results in the adsorption of a large number of phage particles onto the bacterial surface and leads to cell wall destruction (Corbel et al., 1988). On the other hand, an increasing number of phage sequencing results have revealed that there are nucleotide variations in phages with the same-named described by different laboratories. These variations are prone to occur in genes associated with host specificity. Of the five groups of phages, R-group phages are less stable and insensitive to some rough strains (Li and Jiang, 1983), and this could make it difficult to identify this group of *Brucella* phages in the laboratory setting. Irrespective of these differences, there is agreement in the findings with regard to the ability of the bacteriophage NMY-2 isolated from long-term passaged strains in Inner Mongolia to lyse rough *Brucella* strains. Thus, this bacteriophage has high potential for application in *Brucella* typing and identification.

In the experimental procedures, the optimal MOI for phage A1, NMY-1, NMY-2, and Tb_C_ were determined to be 0.1, 0.001, 0.01, and 10, respectively. The MOI represents phage binding to bacteria, however effective adsorption of phage does not necessarily lead to effective infection by phage. We found that the latent and eclipse periods of A1, NMY-2, and the reference phage Tb_C_ were much shorter than those reported in previous studies (Tevdoradze et al., 2015). Moreover, their average burst size was comparatively high. This difference may be related to the different host bacteria used in the studies. In this study, Tb_C_ was incubated with *B. abortus* 544A, while in the previous study, it was incubated with *B. abortus* 141 and *B. abortus* S19 (Tevdoradze et al., 2015). Thus, the incubation period, lysis period, and lytic ability of bacteriophages may differ between host bacteria. This implies that the growth characteristics of bacteriophages are closely related to their host bacteria. If non-standard proliferating host bacteria are used, bacteriophages may be affected by the genetic material of host bacteria and exhibit genetic drift. In addition, the growth status of bacteriophages may also be affected by the characteristics of the phage itself, the host bacteria, and environmental conditions such as the composition of the culture medium and culture temperature. These factors must be considered in phage proliferation experiments. Generally speaking, phages are less stable in highly acidic environments because of protein denaturation, but many phages also survive at pH 3 to 11 (Jamal et al., 2015). In the experimental procedures, phage A1, NMY-1, NMY-2, and Tb_C_ exhibited excellent stability within the pH range of 2 to 13.

Whole-genome sequencing revealed that the genomic features of all phages were similar to those reported for phages such as Wb_W_, Bk_W_, R/C_W_, and EF4 (Cicha et al., 2020; Farlow et al., 2014), and the similarity indicates that these features are highly conserved. In line with this, phylogenetic analysis of the three phages (A1, NMY-1, and NMY-2) isolated in this study showed that they were in the same clade and were closely related to Bk_W_ and distantly related to other *Brucella* bacteriophages. This was reflected in their genome maps, which showed a high degree of similarity. In addition, the host range of A1 and NMY-1 is consistent with that of Bk_W_. This is indicative of a genomic and biological phenotypic correlation. These findings are supported by previous results which have shown that even *Brucella* bacteriophages from different origins are closely related to each other and belong to the same species (Tevdoradze et al., 2015). In fact, comparison of *Brucella* bacteriophages obtained from different origins revealed a high degree of sequence homogeneity between bacteriophage genomes that may be the result of genetic bottlenecks in the *Brucella* bacteriophage population (Farlow et al., 2014; Flores et al., 2012; Tevdoradze et al., 2015). Studies have also shown that most lytic bacteriophages may exhibit more pronounced genomic homogeneity and lower phylogenetic consistency with their bacterial hosts because they are not dependent on host replication systems and have lower transverse genetic exchange frequencies (Chen and Lu, 2002). It was traditionally believed that, despite the capacity of tailed phages to infect identical hosts, tailed phages isolated from distinct geographical locations and different time epochs are unlikely to demonstrate substantial nucleotide similarity (Brüssow and Hendrix, 2002). However, Carrias et al. (2011) indicate that tailed phages sourced from distinct geographical regions can, in fact, display a high level of genomic similarity. The genome of Iz_V_ is the largest of all known *Brucella* phages, and its genome is similar to that of Tb_V_ and Fi_V_. However, according to the phylogenetic tree built based on SNPs, the closest relatives of Iz_V_ are not Tb_M_ or Fi_V_, but the phages Bk2 and R/C_W_. Therefore, sequencing of more and different *Brucella* phages in the future. This effort should cover well-known phages such as Np and other previously isolated ones. Moreover, significantly, newly-discovered environmental phages, which remain under-studied in labs, should also be sequenced. Through this extensive sequencing approach, we may confirm whether the currently observed genetic structure is characteristic of the genome within the *Brucella* bacteriophage family and further broaden our understanding of the genotype–phenotype correlations of *Brucella* phages.

Functional analysis of the genomes of the *Brucella* bacteriophages revealed that the detected genes were mainly related to DNA replication, packaging, and metabolism, as well as host lysis. In line with these findings, it has been reported that *Brucella* phage DNA replication requires a DEAD decarboxylase and a bifunctional enzyme with DNA primase and polymerase activities (Flores et al., 2012). In addition, the putative PolB-associated nucleic acid exonuclease detected may act as an auxiliary protein responsible for DNA primase/polymerase proofreading activity (Gill et al., 2014). Three major proteins involved in DNA packaging were found in the three phages: the terminal enzyme small subunit and large subunits, and the portal protein. Both terminal enzyme subunits remain in the cell and bind to the portal protein to form the DNA packaging motor (Petrovski et al., 2011). In turn, the portal proteins form part of the mature viral particle and can form channels that allow phage DNA to enter and exit the head, thus initiating the assembly of the head and participating in the packaging of the genome (Rao and Feiss, 2008). Two genes that may be involved in DNA restriction and modification were identified in all three phages: a putative DNA methyltransferase and a putative type III restriction nuclease. BLAST searches revealed that methyltransferases share amino acid sequence similarity with members of the NADP-Rossman family of proteins, which span the spectrum of bacteria and phages (Bashton and Chothia, 2002). The same result was observed for type III restriction endonucleases belonging to the PD-(D/E) XK family of proteins (Kinch et al., 2005). These findings suggest that genes involved in DNA metabolism, even those belonging to different lineages, are similar in their putative functions and are fairly common in the genomes of tailed phages (Kinch et al., 2005). Finally, genes encoding for peptidoglycan-binding structural domains and endolysins, which are involved in host lysis, were detected in the phages. Accordingly, it has been found that phages synthesize a cell wall hydrolase, endolysin (Cahill and Young, 2019), in the late stages of bacterial infection that is involved in the hydrolysis of peptidoglycan and release of phage progeny (Marques et al., 2021), capable of degrading peptidoglycan in Gram-negative bacteria (Pei and Grishin, 2005). In addition, consistent with previous observations by Flores et al. (2012), the genomes of these three *Brucella* phages share a high degree of sequence similarity with that of Tb_M_, and two major InDels were identified among the phages.

Comparative analysis of multiple *Brucella* bacteriophages revealed several variations between *Brucella* bacteriophage genes, including the genes encoding putative tail collar proteins, structural proteins, and enzymes. The host range of phages is mainly dependent on the receptor-binding proteins of the phage, the main component of which is the tail-associated lysin proteins (*tal*) (Dowah and Clokie, 2018). The receptor-binding proteins mediate the process of phage attachment to the host bacterial surface receptor and are important for the ability to infect the host (Bertozzi Silva et al., 2016). Thus, changes in the tail collar protein gene and corresponding protein conformational alterations may lead to differences in the host range and lytic ability of *Brucella* bacteriophages. Similar to previous data (Farlow et al., 2014), the present results showed that the gene encoding the putative phage tail collar protein showed the most significant variation. Further, Farlow et al. (2014), Flores et al. (2012), and Tevdoradze et al. (2015) compared the genome-wide sequences of *Brucella* bacteriophages and concluded that the neck collar and tail collar protein genes may be host-specific. In structurally similar T7 phages, Agirrezabala and Aksyuk et al. have shown that conformational changes in the tail collar protein can mediate a complex interaction of bacterial outer membrane penetration and structural transformation of DNA injection channels. They also reported that the function of this protein is to penetrate the bacterial outer membrane, undergo conformational changes in the channel, and release DNA. Therefore, the tail collar protein can be used as a receptor for subsequent studies on the interaction of bacteriophages with host bacteria. In addition to these genes, 7 ORFs in this study (ORF 12, 16, 17, 20, 21, 43, and 58) have also shown genetic variation between *Brucella* phages in other studies (Farlow et al., 2014; Tevdoradze et al., 2015), but variations in the remaining seven (ORFs 2, 13, 14, 19, 30, 46, and 52) have not been reported previously in *Brucella* phages. In addition, variations among ORFs 7, 18, 26, 39, 42, and 51 have been reported (Farlow et al., 2014; Tevdoradze et al., 2015), but no variation in these ORFs was observed among the phages analyzed in this study. Despite the presence of multiple variable sites, further studies are needed to confirm whether they are key factors influencing the phage host range. As there is very little genome-wide data on phages A1, NMY-1, and NMY-2, the data provided here would be highly useful for understanding the composition and classification of *Brucella* bacteriophages. For each bacteriophage, a total of 53 genes were individually predicted, along with their functional annotations and variations. This information is useful for understanding the molecular diversity and attributes of these genes for future comparative studies on the molecular evolution of *Brucella* bacteriophages. This study had some limitations. First, the analysis of the *Brucella* bacteriophages was preliminary; the functions of most of the proteins have not yet been confirmed and must be explored further in the future. Second, more *Brucella* bacteriophages from different hosts and regions should be isolated and compared.

## 5 Conclusion

In summary, the detailed data on the morphology, host range, growth characteristics, physicochemical stability, genome variations, and protein functions of the three phages of *Brucella* phages A1, NMY-1, and NMY-2 provided in this study. These findings lay a solid theoretical foundation for the laboratory culture of *Brucella* phages and their application in the detection and treatment of brucellosis. Specifically, they offer new insights into the development of a novel *Brucella* phage typing system. By enriching the gene pool of *Brucella* phages, the study also provides valuable clues for exploring the genetic evolution of phages and the underlying mechanisms of their interaction with the host. This research not only advances our understanding of *Brucella* phages but also has significant implications for both basic research and potential clinical applications in the context of brucellosis management.

## Data Availability

The accession numbers of phage A1, NMY-1 and NMY-2 in the GenBank database are PP236439, PP236440, and PP236441, respectively.

## Footnotes

^1^ https://blast.ncbi.nlm.nih.gov/Blast.cgi
