# Supplementary material for: Morphological, lytic, and genetic characteristics of three Brucella phages isolated from Inner Mongolia Autonomous Region
Source: Front Microbiol. 2025 Apr 30;16:1550801. doi: 10.3389/fmicb.2025.1550801 (PMC12075418; doi:10.3389/fmicb.2025.1550801)
Supplement: Supplementary file 1 [file Data_Sheet_1.docx]

TABLE S1 Genomic characteristics of *Brucella* phages A1, NMY-1, and NMY-2

| *Brucella* phage | Total base pairs (bp) | Number of genes | GC percent (%) | Taxonomy |
| --- | --- | --- | --- | --- |
| A1 | 38380 | 54 | 48.17 | Caudovirales，Podoviridae |
| NMY-1 | 38380 | 54 | 48.14 | Caudovirales，Podoviridae |
| NMY-2 | 38334 | 54 | 48.18 | Caudovirales，Podoviridae |
