# Supplementary material for: Morphological, lytic, and genetic characteristics of three Brucella phages isolated from Inner Mongolia Autonomous Region
Source: Front Microbiol. 2025 Apr 30;16:1550801. doi: 10.3389/fmicb.2025.1550801 (PMC12075418; doi:10.3389/fmicb.2025.1550801)
Supplement: Supplementary file 2 [file Data_Sheet_2.docx]

TABLE S2 Results of SNP/InDel assays of *Brucella* phages A1, NMY-1, and NMY-2.

| Locus | Annotation | Tb | NMY-1 | NMY-2 | A1 | Strand | ORF |
| --- | --- | --- | --- | --- | --- | --- | --- |
| 708 | hypothetical protein | C | A | A | C | + | 2 |
| 3298 | putative HNH endonuclease | A | A | A | G | + | 8 |
| 7291 | putative portal protein | C | A | A | C | + | 12 |
| 8696 | hypothetical protein | G | T | T | T | + | 13 |
| 9188 | hypothetical protein | . | G | G | G | + | 14 |
| 10105 | structural protein | C | C | C | G | + | 16 |
| 10167 | structural protein | T | G | G | G | + | 16 |
| 11592 | major head protein | A | A | A | G | + | 17 |
| 11610 | major head protein | . | T | T | T | + | 17 |
| 11614 | major head protein | A | . | . | . | + | 17 |
| 12555 | hypothetical protein | C | T | T | T | + | 19 |
| 12556 | hypothetical protein | G | A | A | A | + | 19 |
| 13438 | structural protein | A | G | G | G | + | 20 |
| 13439 | structural protein | T | C | C | C | + | 20 |
| 13440 | structural protein | A | G | G | G | + | 20 |
| 13441 | structural protein | T | C | C | C | + | 20 |
| 13442 | structural protein | A | G | G | G | + | 20 |
| 13443 | structural protein | T | C | C | C | + | 20 |
| 13449 | structural protein | C | T | T | T | + | 20 |
| 13450 | structural protein | C | T | T | T | + | 20 |
| 15774 | structural protein | C | C | C | A | + | 21 |
| 15916 | structural protein | C | T | T | T | + | 21 |
| 16700 | structural protein | C | T | T | C | + | 23 |
| 17184 | structural protein | G | . | . | . | + | 23 |
| 17185 | structural protein | G | . | . | . | + | 23 |
| 17186 | structural protein | T | . | . | . | + | 23 |
| 17187 | structural protein | A | . | . | . | + | 23 |
| 17188 | structural protein | G | . | . | . | + | 23 |
| 17189 | structural protein | C | . | . | . | + | 23 |
| 18109 | putative peptidoglycan hydrolase | C | C | C | T | + | 25 |
| 21792 | putative tail collar protein | C | A | A | A | + | 27 |
| 21824 | putative tail collar protein | C | A | A | A | + | 27 |
| 21955 | putative tail collar protein | C | A | A | A | + | 27 |
| 22364 | putative tail collar protein | G | T | T | T | + | 27 |
| 22409 | putative tail collar protein | G | T | G | T | + | 27 |
| 22422 | putative tail collar protein | C | C | . | C | + | 27 |
| 22423 | putative tail collar protein | A | A | . | A | + | 27 |
| 22424 | putative tail collar protein | A | A | . | A | + | 27 |
| 22425 | putative tail collar protein | T | T | . | T | + | 27 |
| 22426 | putative tail collar protein | A | A | . | A | + | 27 |
| 22427 | putative tail collar protein | C | C | . | C | + | 27 |
| 22466 | putative tail collar protein | C | A | G | A | + | 27 |
| 22470 | putative tail collar protein | A | G | A | G | + | 27 |
| 22473 | putative tail collar protein | A | A | C | A | + | 27 |
| 22474 | putative tail collar protein | T | T | A | T | + | 27 |
| 22476 | putative tail collar protein | G | G | T | G | + | 27 |
| 22478 | putative tail collar protein | C | C | A | C | + | 27 |
| 22481 | putative tail collar protein | G | A | G | A | + | 27 |
| 22493 | putative tail collar protein | A | A | G | A | + | 27 |
| 25598 | putative endolysin | T | G | T | T | + | 30 |
| 25741 | putative endolysin | G | G | A | G | + | 30 |
| 25905 | putative endolysin | G | G | T | G | + | 30 |
| 29915 | putative DNA methyl transferase | C | A | A | A | - | 43 |
| 31658 | hypothetical protein | C | C | A | C | - | 46 |
| 35637 | putative DNA-PolB associated exonuclease | G | A | G | G | - | 52 |
| 39116 | putative bifunctional DNA primase/polymerase | G | G | G | T | - | 58 |
| 39320 | putative bifunctional DNA primase/polymerase | A | C | C | C | - | 58 |
| 40845 |  | G | T | T | T | - |  |
| 40857 |  | T | C | T | T | - |  |
